# Effectiveness of Community versus Hospital Eye Service follow-up for patients with neovascular age-related macular degeneration with quiescent disease (ECHoES): a virtual non-inferiority trial

**DOI:** 10.1136/bmjopen-2015-010685

**Published:** 2016-07-01

**Authors:** Barnaby C Reeves, Lauren J Scott, Jodi Taylor, Simon P Harding, Tunde Peto, Alyson Muldrew, Ruth E Hogg, Sarah Wordsworth, Nicola Mills, Dermot O'Reilly, Chris A Rogers, Usha Chakravarthy

**Affiliations:** 1 Clinical Trials and Evaluation Unit, School of Clinical Sciences, University of Bristol, Bristol, UK; 2 Department of Eye and Vision Science, Institute of Ageing and Chronic Disease, University of Liverpool, Liverpool, UK; 3 NIHR BMRC at Moorfields Eye Hospital NHS Foundation Trust and UCL Institute of Ophthalmology, London, UK; 4 Centre for Experimental Medicine, Queen's University Belfast, Belfast, UK; 5 Health Economic Research Centre, Nuffield Department of Population Health, University of Oxford, Oxford, UK; 6 School of Social and Community Medicine, University of Bristol, Bristol, UK; 7 Centre for Public Health, Queen's University Belfast, Belfast, UK

**Keywords:** AMD, Wet AMD Reactivation, Optical Coherence Tomography

## Abstract

**Objectives:**

To compare the ability of ophthalmologists versus optometrists to correctly classify retinal lesions due to neovascular age-related macular degeneration (nAMD).

**Design:**

Randomised balanced incomplete block trial. Optometrists in the community and ophthalmologists in the Hospital Eye Service classified lesions from vignettes comprising clinical information, colour fundus photographs and optical coherence tomographic images. Participants' classifications were validated against experts' classifications (reference standard).

**Setting:**

Internet-based application.

**Participants:**

Ophthalmologists with experience in the age-related macular degeneration service; fully qualified optometrists not participating in nAMD shared care.

**Interventions:**

The trial emulated a conventional trial comparing optometrists' and ophthalmologists' decision-making, but vignettes, not patients, were assessed. Therefore, there were no interventions and the trial was virtual. Participants received training before assessing vignettes.

**Main outcome measures:**

Primary outcome—correct classification of the activity status of a lesion based on a vignette, compared with a reference standard. Secondary outcomes—potentially sight-threatening errors, judgements about specific lesion components and participants' confidence in their decisions.

**Results:**

In total, 155 participants registered for the trial; 96 (48 in each group) completed all assessments and formed the analysis population. Optometrists and ophthalmologists achieved 1702/2016 (84.4%) and 1722/2016 (85.4%) correct classifications, respectively (OR 0.91, 95% CI 0.66 to 1.25; p=0.543). Optometrists' decision-making was non-inferior to ophthalmologists' with respect to the prespecified limit of 10% absolute difference (0.298 on the odds scale). Optometrists and ophthalmologists made similar numbers of sight-threatening errors (57/994 (5.7%) vs 62/994 (6.2%), OR 0.93, 95% CI 0.55 to 1.57; p=0.789). Ophthalmologists assessed lesion components as present less often than optometrists and were more confident about their classifications than optometrists.

**Conclusions:**

Optometrists' ability to make nAMD retreatment decisions from vignettes is not inferior to ophthalmologists' ability. Shared care with optometrists monitoring quiescent nAMD lesions has the potential to reduce workload in hospitals.

**Trial registration number:**

ISRCTN07479761; pre-results registration.

**Strengths and limitations of this study**

- The virtual design allowed us to do the trial quickly and efficiently, recruiting ophthalmologists and optometrists from across the UK; vignettes were created from information for actual patients, collected for a randomised trial.
- Virtual decision-making is different from face-to-face decision-making in a clinic but similar to how some hospitals are managing their workload.
- Optometrists would have liked more training but nevertheless achieved non-inferior performance with respect to the prespecified inferiority margin.
- Vignettes were created using images obtained with spectral domain systems that afforded poorer quality visualisation than currently.
- Groups of ophthalmologists and optometrists had different average durations since obtaining the qualification that made them eligible to take part but were trained identically.

## Introduction

Wet or neovascular age-related macular degeneration (nAMD) is a common eye condition that causes severe sight loss and blindness. Lesions develop due to new blood vessels growing from the choroid or within the retina, which breach the normal tissue barriers and come to lie within the subpigment epithelial and subretina. nAMD is treated by intravitreal injections of antivascular endothelial growth factor drugs,^1^ which inhibit vascular endothelial growth factor, until the lesion becomes dormant. However, dormant lesions can reactivate, and therefore, patients need to be monitored at regular hospital visits. These visits involve visual acuity checks, clinical examination and optical coherence tomograms (OCT). Treatment is restarted if a lesion has reactivated.

Regular monthly review in the UK Hospital Eye Service (HES) blocks clinic space, uses valuable resources, is expensive and is also burdensome to the patients and their carers. The total number of patients with nAMD requiring regular monitoring has not yet reached a steady state because, based on current experience, monitoring needs to continue until vision becomes so poor that a patient declines to attend or dies. Therefore, some hospitals are struggling to provide clinic appointments at recommended intervals. This situation has prompted service providers to explore innovative models of service provision.

‘Shared care’ between optometrists in private practice in the community and the HES is reasonably well established for eye conditions other than nAMD, such as glaucoma.^2^ ^3^ A recent review^4^ showed a number of scenarios used to increase the capacity of nAMD services across the UK; many scenarios involved extended roles for optometrists and nurse practitioners but only when working within the HES. Other studies have evaluated the potential of capturing OCTs in an outreach service, but these still involve assessments by ophthalmologists working in the HES.^5^ ^6^

The Effectiveness of Community versus Hospital Eye Service follow-up for patients with quiescent nAMD (ECHoES) trial was designed to test the hypothesis that, after uniform training, decisions about the reactivation of nAMD lesions previously classified as quiescent made by optometrists working in the community are not inferior to decisions made by ophthalmologists working in the HES.

## Methods

### Study design

The ECHoES trial is a non-inferiority trial designed to emulate a parallel group design. Decisions about the reactivation status of lesions were made from vignettes, consisting of sets of retinal images (colour and spectral domain OCT) with accompanying clinical information, rather than by examining actual patients. Retreatment decision-making on the basis of review of images, in the absence of the patient, is a strategy that is increasingly being used by the HES to improve the efficiency of nAMD clinics. A NHS Research Ethics committee gave a favourable ethical opinion of the study on 14 May 2013 (reference 13/EM/0199). The trial is registered (ISRCTN07479761; pre-results registration). The study sponsor was the Queen's University Belfast. The study was conducted in accordance with the principles of the International Conference on Harmonisation of Good Clinical Practice under the oversight of the study sponsor.

Ninety-six participants (48 optometrists and 48 ophthalmologists) each assessed 42 vignettes. A randomised balanced incomplete block design was used,^7^ ^8^ with each vignette assessed by seven participants in each professional group. A secure web-based application was developed to allow participants to take part in the trial remotely; a demonstration can be viewed at http://www.echoestrial.org/demo/Account/Register.aspx. Further details have been described elsewhere (http://www.nets.nihr.ac.uk/__data/assets/pdf_file/0007/81196/PRO-11-129-195.pdf; accessed 19 November 2015).^9^

### Vignettes

A database containing 288 vignettes was created from the clinical and image repository of a previously conducted clinical trial (HTA ref.: 07/36/01; ISRCTN92166560; pre-results registration^10^ ^11^); this number of vignettes is explained below. The vignette consisted of a brief clinical summary that provided a patient's age, gender, cardiovascular health and smoking status; two sets of images comprising colour fundus and radial pattern spectral domain OCT from two separate visits with the corresponding visual acuity from each visit. The two sets of images were termed baseline and index, with the former from a visit when the lesion was quiescent and the latter from a visit when the lesion could have been either quiescent or reactivated.

### Participants

The ECHoES trial recruited ophthalmologists and optometrists working in the UK, through information circulated in optometry journals and forums and to ophthalmologists' email lists. Ophthalmologists were required to have 3 years’ postregistration experience in ophthalmology, have passed the part 1 examination of the Royal College of Ophthalmologists or the Diploma in Ophthalmology or equivalent and have experience within the AMD service (no minimum duration specified). Optometrists were required to be fully qualified, registered with the General Optical Council for at least 3 years and not be participating or have participated in any AMD shared care scheme.

### Training

All participants received the same training. Ophthalmologists and optometrists are qualified to detect retinal pathology, but optometrists may not have the skills to detect lesion reactivation. Although OCTs are increasingly being incorporated into optometry practices, this would have been a completely new imaging modality for many. Eligible ophthalmologists may also not have been fully trained to detect lesion reactivation since doctors without specialist skills (grade ST1 and above) often staff retina clinics in the HES.

There were two aspects of training. First, participants had to attend two online webinars; second, each participant had to assess a set of training vignettes and achieve a criterion level of performance. The webinars provided a reproducible package of background to the trial, information about the management of nAMD and how to assess OCT and colour retinal images for relevant features and the trial. Each participant assessed 24 training vignettes, randomly sampled from the vignette database after excluding any vignette preassigned for the participant's main trial assessments. The pass mark was set at 75% (18 of 24); if this was not attained, a second attempt was permitted with different training vignettes, but participants who failed to achieve the pass mark a second time were withdrawn from the trial. Further information, including details of the content of the lectures, is available elsewhere.^9^

### Outcomes

The primary outcome was correct classification of the activation status of the nAMD lesion characterised in the vignette at the index visit, from the images and other information the vignette contained. Participants' classifications (reactivated, quiescent or suspicious) were judged against an expert reference standard (see below). For the primary outcome, suspicious and quiescent classifications were grouped, creating a binary outcome (reactivated vs suspicious/quiescent). A participant's lesion classification was scored as correct if the participant's classification and the reference standard lesion classification matched, that is, both were ‘reactivated’, or both were suspicious/quiescent.

Secondary outcomes were as follows: the frequency of potentially sight-threatening errors (defined as a ‘reactivated’ vignette, based on the reference standard, classified as ‘quiescent’ by a participant); participants' judgements about the presence or absence, and increase from baseline, of lesion components (subretinal fluid, intraretinal cysts, diffuse retinal thickening (DRT), pigment epithelial detachment (PED), blood and exudates); participant-rated confidence in their decisions about the primary outcome on a five-point scale; and cost-effectiveness of monitoring patients with quiescent lesions by optometrists in the community compared to ophthalmologists in the HES. In addition, we carried out focus groups to ascertain the views of patients, their representatives, optometrists, ophthalmologists and clinical commissioners on the proposed shared care model. The results of the cost-effectiveness analysis and focus groups are not described in this article.

We believed that the training would be very important in order for participants to achieve good performance when assessing the vignettes. Therefore, in order to help interpret the results, we emailed a questionnaire to all participants (n=102) who completed training to obtain feedback about their perceptions of the quality (6-point scale: excellent, very good, good, fair, poor and very poor) and adequacy of the training (3-point scale: completely sufficient, additional training required and completely different training required).

### Reference standard

A reference standard lesion classification was established for each vignette based on the judgements of three medical retina experts (UC, SPH and TP; these experts lead the UK Network of Ophthalmic Reading Centres). The experts agreed a framework for assigning a reference classification (table 1), which meant that disagreements between experts could only arise from disagreements about the presence or absence of specific features, not from disagreements in how the features were interpreted. Using the web-based application, the experts independently assessed the image features without reference to participants' assessments and classified all 288 vignettes as quiescent, suspicious or reactivated. A meeting was then held to establish a consensus classification for 69 vignettes for which experts' independent classifications disagreed.

**Table 1:** Framework for reference classifications

Feature	Lesion reactivated	Lesion quiescent
SRF on OCT	Yes	No
	*Or*	*And*
IRC on OCT	Yes, *and* increased from baseline	No/not increased from baseline
	*Or*	*And*
DRT on OCT	Yes, *and* increased from baseline	No/not increased from baseline
	*Or*	*And*
Blood on CF	Yes, *and* increased from baseline	No/not increased from baseline
	*Or*	*And*
Exudates on CF	Yes, *and* increased from baseline	No/not increased from baseline

CF, colour fundus; DRT, diffuse retinal thickening; IRC, intraretinal cysts; OCT, optical coherence tomogram; SRF, subretinal fluid.

During participants' training, it was explained that the presence or absence of certain features indicated the activity status of a lesion; the experts' framework defined how combinations of features related to the overall assessment of lesion activity. A set of validation rules based on the framework was applied to the assessment system. So, when participants entered data into the web application,^9^ the system displayed a message if the combination of responses and the chosen activity status was not consistent with the rules. Participants were asked to confirm their responses (despite the inconsistency) or edit them.

### Statistical analysis

We set a non-inferiority limit of 10%, assuming that ophthalmologists would correctly classify at least 95% of their vignettes; this limit and assumption correspond to an OR of 0.298. We assumed that 10% poorer performance would be the maximum difference that would be acceptable. The choice of margin was not based on prior evidence but was appraised by five peer-reviewers, none of whom suggested it was too large.

The sample size was estimated as recommended by Lu and Bean.^12^ The calculation gave an unconditional maximum sample size of 266 to have 90% power to test the non-inferiority hypothesis with 2.5% (one-sided) statistical significance. This number was increased to 288 to ensure we achieved balance in the design (ie, each vignette assessed the same number of times, with each assessor reviewing the same number of vignettes). We considered lower values for the proportion correctly classified by ophthalmologists; providing ophthalmologists classified at least 75% of vignettes correctly, the sample size was sufficient to test the hypothesis for a margin no greater than 7.5%. We assumed for this calculation that each vignette would only be assessed once by each professional group; in fact, each vignette was seen by seven participants in each group, so the trial had 90% power to detect non-inferiority for a lower proportion of vignettes correctly classified by ophthalmologists.

The analysis population was all participants who successfully completed training and assessed all their main study vignettes. Continuous variables are summarised by means and SDs, or medians and IQRs if distributions were skewed. Categorical data are summarised as a number and percentage. Baseline participant characteristics are described and groups formally compared using t tests, Mann-Whitney tests, χ^2^ tests or Fishers exact tests as appropriate.

All primary and secondary outcomes were analysed using mixed effects regression models, adjusting for the order in which the vignettes were viewed as a fixed effect (tertiles: 1–14, 15–28 and 29–42) and participant and vignette as random effects. All outcomes were binary and analysed using logistic regression with group estimates presented as ORs with 95% CIs. For hypothesis tests, two-tailed p values <0.05 were considered statistically significant; likelihood ratio tests were used in preference to Wald tests.

There were no changes to the methods for the study. However, the questionnaire about participants' perceptions of training was added shortly after starting to recruit and the methods of analysis were changed to accommodate the fact that experts classified some lesions as suspicious.

## Results

### Recruitment

**Figure 1:**
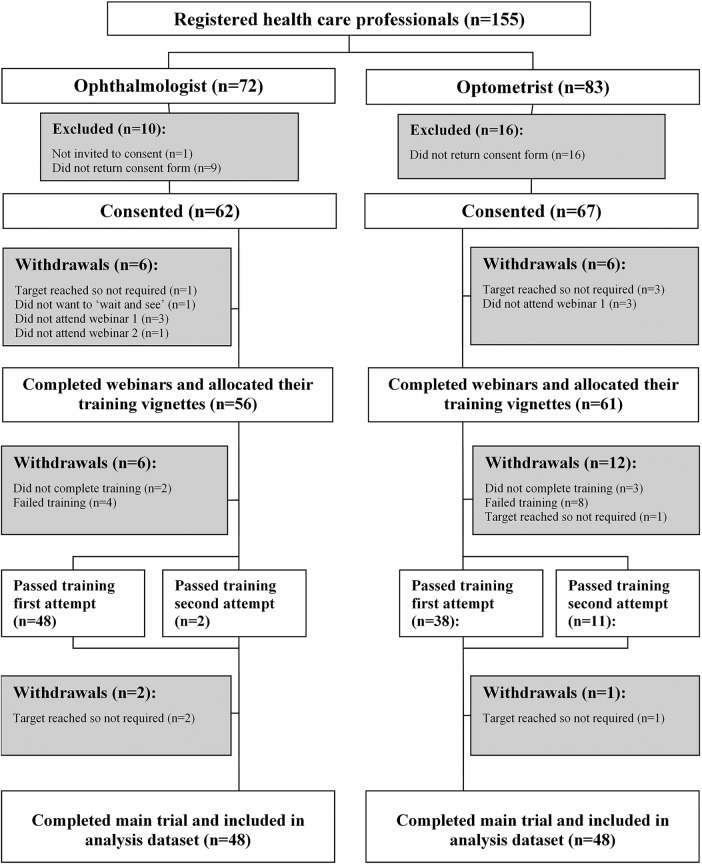
Flow of participants. At the start of the trial, we were unsure how many participants we would need to recruit in order to meet our target of 48 participants in each group. Therefore, we over-recruited at the consent stage and asked a number of participants to complete the webinar and then ‘wait and see’ whether we needed them to participate in the main trial. We also slightly over-recruited at each stage of the trial to account for withdrawals. This resulted in a small number of participants being withdrawn at various stages of the trial because they were no longer required.

Between 1 June 2013 and 6 March 2014, 155 healthcare professionals registered their interest in the trial, and of these, 62 ophthalmologists and 67 optometrists consented to take part. A number of participants withdrew or were withdrawn due to not completing their webinar training, not achieving the required performance level in their training vignettes, no longer being required or no longer wishing to take part (figure 1). Of 56 ophthalmologists who started to assess training vignettes, 2 did not complete their assessments of training vignettes and 4 failed to achieve the pass mark twice and were not allowed to continue in the trial; 48 of the remaining 50 achieved the pass mark with their first set of training vignettes and 2 with their second set. Of 61 optometrists who started to assess training vignettes, 4 did not complete their assessments of training vignettes (1 because the target number of optometrists had been attained) and 8 failed to achieve the pass mark twice and were not allowed to continue in the trial; 38 of the remaining 49 achieved the pass mark with their first set of training vignettes and 11 with their second set. As planned, 48 ophthalmologists and 48 optometrists completed the full trial and formed the analysis population.

### Reference standard classifications

The reference standard classifications for the 288 vignettes were: 142 reactivated (49.3%), 141 (49.0%) quiescent and 5 (1.7%) suspicious.

### Participant characteristics

The demographic characteristics of optometrists and ophthalmologists were similar (mean (SD) age 43.1 (10.1) and 42.2 (8.0) years, respectively, p=0.61; 50.0% vs 43.8% were women, p=0.54). Optometrists had more years of qualified experience than ophthalmologists (median (IQR) 17.4 (10.1 to 28.4) and 11.4 (4.8 to 16.9) years, p<0.001).

### Primary outcome

**Figure 2:**
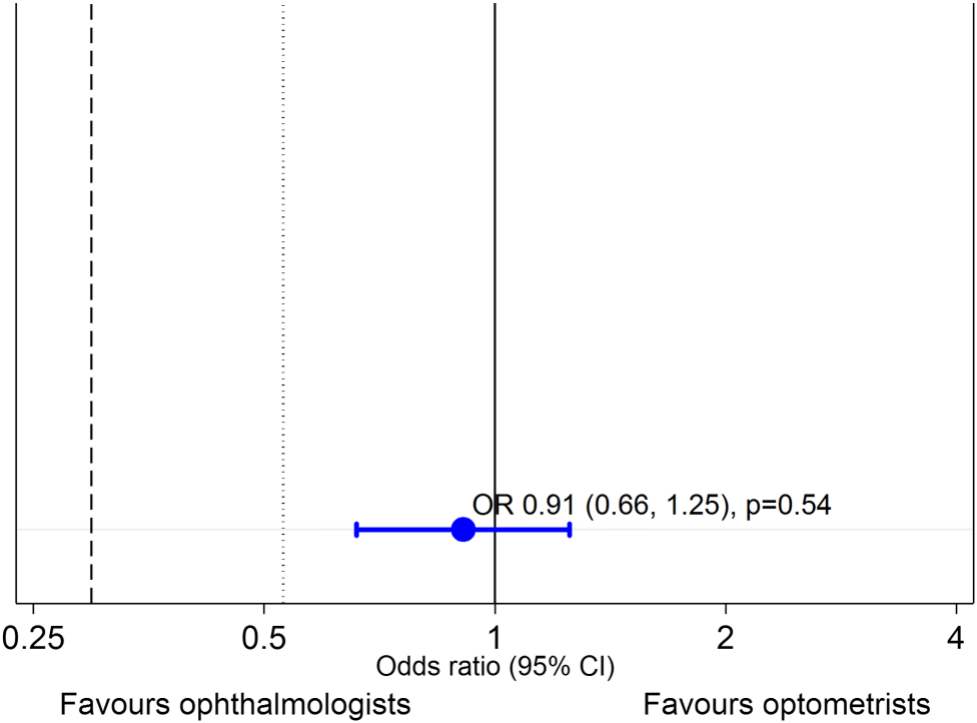
Comparison between optometrists and ophthalmologists for the primary outcome. The point estimate for the effect size comparing optometrists with ophthalmologists (reference category) for the outcome of correct classification is shown as an odds ratio (OR) and the error bar represents the 95% CI. (The OR of 0.91 means that optometrists had slightly lower odds of making a correct classification than ophthalmologists, hence the treatment effect slightly ‘favours’ ophthalmologists.) The line of no difference is illustrated by the solid vertical line at 1. The black dashed line (at 0.30) represents the prespecified non-inferiority limit of 10% poorer performance (assuming 95% of vignettes would be classified correctly by ophthalmologists). The black dotted line (at 0.53) represents the same non-inferiority limit of 10% but for the observed percentage of vignettes classified correctly by ophthalmologists (85%).

Ophthalmologists and optometrists correctly classified the nAMD lesion in the index images of 1722/2016 (85.4%) and 1702/2016 (84.4%) vignettes. The median number of correct lesion classifications among ophthalmologists was 37/42 (IQR 35.0–38.5, minimum 26, maximum 41) and, among optometrists, was 36/42 (IQR 33.0–38.0, minimum 24, maximum 41). The ability of optometrists to classify lesions correctly was non-inferior (clinically and statistically) to the ability of ophthalmologists, according to the prespecified limit of 10% absolute difference (dotted black line in figure 2). In this primary outcome model, the variance attributed to the participant random effect was far smaller than that of the vignette random effect (0.360 vs 2.062, respectively). Participants over-ruled the validation framework for 2.5% of all assessments (102/4032).

**Figure 3:**
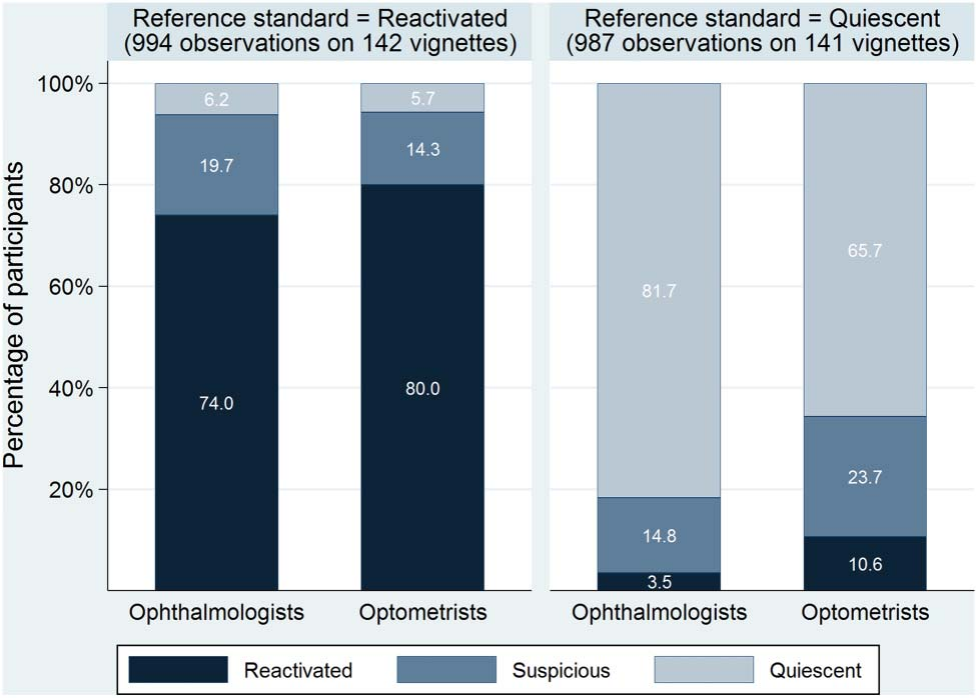
Participants' lesion classifications for vignettes classified as reactivated or quiescent by the reference standard. Thirty-five observations in each group, for the five vignettes classified by the reference standard as ‘suspicious’ which were each assessed by seven professionals, are not shown in this graph. (The data are as follows—ophthalmologists: 1=Reactivated (2.9%), 17=Suspicious (48.6%), 17=Quiescent (48.6%); optometrists: 10=Reactivated (28.6%), 11=Suspicious (31.4%), 14=Quiescent (40.0%)). These data are consistent with optometrists making more cautious retreatment decisions than ophthalmologists.

Optometrists were more likely to correctly classify a vignette as reactivated than ophthalmologists (795/994 (sensitivity=80.0%) vs 736/994 (sensitivity=74.0%)), but were less likely to correctly classify a vignette as quiescent/suspicious (907/1022 (specificity=88.7%) vs 986/1022 (specificity=96.5%); figure 3). A post hoc analysis quantified this interaction (χ^2^=50.4, df 1, p<0.001) between professional group and reference standard classification (reactivated vs quiescent/suspicious); the odds of an optometrist being correct was ∼50% higher than an ophthalmologist if the reference standard classification was reactivated (OR 1.52, 95% CI 1.08 to 2.15; p=0.018), but ∼70% lower if the reference standard classification was quiescent/suspicious (OR 0.27, 95% CI 0.17 to 0.44; p<0.001).

### Secondary outcomes

Serious sight-threatening errors could only occur for the vignettes that were classified as reactivated by the reference standard. These errors occurred in 62/994 (6.2%) of ophthalmologists' classifications and 57/994 (5.7%) of optometrists' classifications (OR 0.93, 95% CI 0.55 to 1.57; p=0.789).

**Figure 4:**
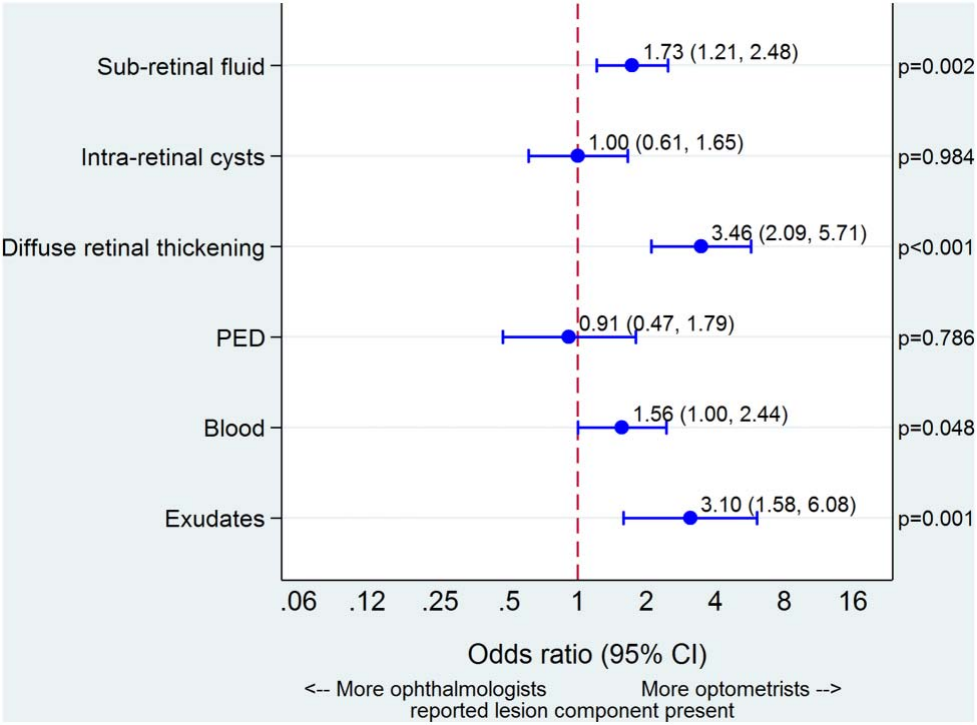
Reporting of lesion components as present. Point estimates for different lesion components are odds ratios and error bars are 95% CIs. Line of no difference is illustrated by the vertical dashed line at 1. PED: pigment epithelial detachment.

The three experts did not attempt to reach consensus about the presence of lesion components in a vignette. Therefore, participants' responses about the presence of lesion components were compared by professional group, without relating these to a reference standard. Optometrists judged lesion components to be present more often than ophthalmologists for all components except PED, consistent with their higher overall sensitivity. This difference was particularly evident for DRT and exudates (table 2; figure 4).

**Table 2:** Secondary outcomes of sight-threatening errors, identification of lesion features and confidence ratings

	Ophthalmologists (n=48)		Optometrists (n=48)		OR (95% CI)	p Value
Secondary outcome	n	Per cent	n	Per cent		
Sight-threatening errors	62/994	6.2	57/994	5.7	0.93 (0.55 to 1.57)	0.789
Is there SRF?	515/2016	25.5	627/2016	31.1	1.73 (1.21 to 2.48)	0.002
Has it increased since baseline?	498/515	96.7	541/627	86.3		
Are there IRC?	799/2016	39.6	808/2016	40.1	1.00 (0.61 to 1.65)	0.985
Has it increased since baseline?	667/799	83.5	683/808	84.5		
Is there DRT?	482/2016	23.9	826/2016	41.0	3.46 (2.09 to 5.71)	<0.001
Has it increased since baseline?	381/482	79.0	597/826	72.3		
Is there any PED?	845/2016	41.9	842/2016	41.8	0.91 (0.47 to 1.79)	0.786
Has it increased since baseline?	311/845	36.8	392/842	46.6		
Is there blood?	150/2016	7.4	194/2016	9.6	1.56 (1.00 to 2.44)	0.048
New or increased since baseline?	126/150	84.0	146/194	75.3		
Are there exudates?	152/2016	7.5	380/2016	18.8	3.10 (1.58 to 6.08)	<0.001
New or increased since baseline?	38/152	25.0	87/380	22.9		
Confidence rating						
1	7/2016	0.3	52/2016	2.6		
2	26/2016	1.3	140/2016	6.9		
3	220/2016	10.9	496/2016	24.6	0.15 (0.07 to 0.32)*	<0.001
4	588/2016	29.2	753/2016	37.4		
5	1175/2016	58.3	575/2016	28.5		
Correct lesion classifications for each confidence rating†						
1	3/7	42.9	42/52	80.8		
2	21/26	80.8	114/140	81.4		
3	147/220	66.8	362/496	73.0		
4	474/588	80.6	634/753	84.2		
5	1077/1175	91.7	550/575	95.7		

*Comparison of optometrists versus ophthalmologists; odds of a confidence rating of 5 versus a rating of 1–4.

†For example, of the seven vignettes to which ophthalmologists gave a confidence rating of 1, three were correct with respect to the primary outcome.

DRT, diffuse retinal thickening; IRC, intraretinal cysts; OR, odds ratio; PED, pigment epithelial detachment; SRF, subretinal fluid.

Ophthalmologists were more confident about their classifications than optometrists; ophthalmologists were very confident (5 on the rating scale) for 1175/2016 (58.3%) vignettes, whereas optometrists had a similar level of confidence for only 575/2016 (28.5%) vignettes (OR 0.15, 95% CI 0.07 to 0.32; p<0.001). For both groups, over 90% of very confident classifications were correct, but there was no clear relationship between confidence and correctness for less confident ratings.

No harms could arise in the trial because decisions were being made for vignettes.

Among the respondents (92/102, 90%) to the questionnaire about training, the quality of the training was perceived to be good, very good or excellent by 90% of ophthalmologists (43/47) and 70% of optometrists (39/55). The majority of ophthalmologist respondents (70%) considered that the training was completely sufficient compared to 11% of optometrists.

## Discussion

### Main findings

Optometrists were non-inferior to ophthalmologists with respect to the overall proportion of lesions correctly classified, but they made more cautious decisions. Compared to ophthalmologists, they were less likely to classify a reactivated lesion as quiescent or suspicious and more likely to classify a quiescent or suspicious lesion as reactivated. More cautious decision-making may be desirable, since it minimises the risk of false-negative misclassifications; furthermore, it is consistent with community optometrists' obligation under their service contract to refer any suspected pathology, although it limits the potential for community monitoring to reduce the HES workload and be cost-effective. The finding may also reflect optometrists having more difficulty in interpreting the diversity of appearance of quiescent lesions, that is, eyes with an abnormal appearance but not needing treatment.

The virtual nature of the trial was certainly efficient compared to a conventional trial recruiting patients to shared or conventional care. Recruitment of all participants, provision of training and assessment of all vignettes took just under 11 months. We suspect that a conventional trial to address the research question would not have been feasible, regardless of the time or expense, since the focus groups indicated that participants might have perceived shared care to be more risky.^9^ ^12^ It would also have had to recruit ∼500 participants to have similar power for the observed success rate of 85% and a non-inferiority margin of 10%.

The staged nature of the trial and the risk of withdrawals, combined with the priority of maintaining the balanced incomplete blocks design, made it difficult to manage recruitment efficiently. The limited number of vignettes, which required us to sample training vignettes from among the 288 vignettes used for the trial assessments, meant that each participant had to be assigned 42 specific vignettes (according to the blocked design) for the main trial before starting training. This constraint prevented a standby participant from progressing in the trial until an existing participant had definitively withdrawn and freed up a specified set of vignettes.

The difference between professions in the overall proportion of vignettes correctly classified will vary depending on the proportion of vignettes classified as active by the reference standard because optometrists tended to make more false-positive referrals. Therefore, we considered whether the proportion of active vignettes in the study was representative. In the IVAN trial, the lesion was judged to be active among participants allocated to discontinuous treatment at 30% of visits in year 1 and 27% in year 2; the median percentage within patients in both years was 33%. Although these percentages are lower than in the ECHoES vignette database, there are two reasons why the percentage may well be higher in real life than in the IVAN trial. First, active lesions in the IVAN trial were identified using either Stratus or Fourier domain OCT equipment; detecting activity with the former equipment is more difficult and is likely to have underestimated the proportion of active lesions. Second, the treatment-as-needed regimen was probably more intensive in the IVAN trial (three treatments at monthly intervals were mandated) than in current usual care.

### Strengths and limitations

The fact that vignettes were created from information for actual patients, documented from diagnosis in the context of a randomised trial, is a key strength but also caused limitations (see below). The web application allowed us to recruit ophthalmologists and optometrists from across the UK and to carry out the trial quickly and efficiently.

The two main limitations of our study are the virtual nature of the trial and the adequacy of training. Decision-making on the basis of a vignette is different from face-to-face decision-making in a clinic. However, decision-making on the basis of investigations made previously, in the absence of the patient, is similar to how some hospitals are managing their workload. More optometrists than ophthalmologists had to assess two sets of training vignettes to attain the pass mark (in effect, additional training), but the results of the main trial suggest that the combination of webinars and sets of training vignettes was sufficient to enable the optometrists to classify lesion activity as well as the ophthalmologists. Feedback questionnaires about training showed most respondents thought that the quality was good but that optometrists would have liked more training (consistent with more optometrists needing to assess two sets of training vignettes). The online platform used for the trial is well suited to delivering more training, ‘refreshers’ and, potentially, interaction with experts about images considered to be especially difficult.

We were limited by the types of OCT scan that were available as these images were acquired during the trial using equipment and a protocol and that were optimal at the time. Current spectral domain systems have improved software for the visualisation of images, which also permit alignment and comparison of scans from different visits. Therefore, clinicians in the HES may have been disadvantaged if they were accustomed to interpreting signs of lesion activity or quiescence using more scans than we were able to display in a vignette.

There are clear differences between ophthalmologists and optometrists in their training, exposure to retinal diseases and imaging technologies, which we considered when defining the eligibility criteria for participants. Trainee ophthalmologists are exposed to retinal services and imaging of retinal anatomy throughout their training period; all NHS clinics in the UK are now equipped with high-resolution Fourier domain OCT systems, and these are used extensively in the diagnosis and management of all posterior ocular segment disorders. Accepting that the qualifications implied different knowledge and experience and might be acquired at varying age/maturity, we therefore specified that participants for both professions must have had 3 years' post qualification experience. We do not think that the difference between professions in the median number of years' experience (after obtaining the qualification that confirmed eligibility) affected the findings. We also trained ophthalmologists and optometrists identically.

There was a small difference between professional groups in the proportions of participants who failed to achieve the required standard to progress to the main trial. These numbers are small and the difference is uncertain. Moreover, implementation of shared care would require a rigorous standard to be applied, as was the case in our study; what matters with respect to implementation is that a rigorous standard should be set, at least as rigorous as adopted for this study (since we showed that optometrists who passed the standard for progressing to the main trial performed as well as the ophthalmologists who passed the standard).

### The trial findings in the context of existing evidence

There is currently no evidence about the effectiveness of community follow-up by optometrists for nAMD. However, there is evidence about the effectiveness of optometrists in providing ‘shared care’ with the HES for glaucoma and diabetic eye disease and the associated training programmes. Evaluations comparing management by optometrists and ophthalmologists have shown acceptable levels of agreement between the decisions made in the context of glaucoma and accident and emergency services.^2^ ^3^ A recent review of approaches to increase the capacity of nAMD services in the UK described scenarios involving extended roles for optometrists and nurse practitioners but only within the HES.^4^ Other studies have evaluated the potential of decision-making in the patient's absence using OCTs captured by outreach services but have only studied ophthalmologists' decisions.^5^ ^6^

Taking and interpreting retinal images are skills that can be easily taught (the former is usually carried out by technicians in the HES), and therefore, the final evaluation in a telemedicine scenario need not always involve an ophthalmologist. Many hospitals already involve specialist optometrists and nurse practitioners in making clinical decisions, although the effectiveness of these management pathways has not been formally evaluated. Shared care in the manner envisaged in this trial requires the ability to interpret signs in the colour and OCT retinal images and the facility for patients to be returned urgently to the HES when reactivation is detected. Such ability needs to be verified and regularly updated for quality assurance.

Qualitative research carried out alongside the main trial identified potential barriers to implementation, including ophthalmologists' and service users' perceptions of optometrists' competence and the need for excellent collaborative working between optometrists and ophthalmologists with respect to managing urgent referrals.^13^ Despite these challenges, optometrists represent a highly skilled and motivated workforce in the UK, the vast majority of whom work in the community. Many optometric practices have invested in the equipment required to obtain the necessary retinal images and already use these to inform their day-to-day decisions. An online platform such as that used for this trial could be used to provide quality assurance during implementation of shared care and initial referral decisions could be mentored or made jointly by optometrist and ophthalmologist to foster confidence. A cost-effectiveness analysis was also carried out alongside the trial and will be reported separately.

### Conclusions

The ECHoES study has demonstrated that, after appropriate training, community-based optometrists were as good as HES ophthalmologists in classifying the activity status of a lesion, but tended to make different types of errors. The tendency of optometrists to be more cautious than ophthalmologists, being less likely to misclassify reactivated lesions but more likely to classify quiescent lesions as reactivated, may be desirable in a primary care setting.

The barriers to implementing a shared care policy based on the model evaluated in ECHoES (above) could be addressed by implementing continuous quality assurance of the performance of optometrists and rapid referral to HES alongside shared care. We do not believe that further research involving patient participants, for example, a conventional trial, is required. A web platform such as the one developed for the ECHoES study could provide additional training and practice in judging vignettes and quality assurance of the performance of optometrists. Hospital information systems routinely provide data to audit the timeliness of other services (eg, cancer waiting times^14^) and it should be possible to adapt them to audit rapid referral.
